# Heterologous prime-boost-boost immunisation of Chinese cynomolgus macaques using DNA and recombinant poxvirus vectors expressing HIV-1 virus-like particles

**DOI:** 10.1186/1743-422X-8-429

**Published:** 2011-09-07

**Authors:** Simon H Bridge, Sally A Sharpe, Mike J Dennis, Stuart D Dowall, Brian Getty, Donald S Anson, Michael A Skinner, James P Stewart, Tom J Blanchard

**Affiliations:** 1 Clinical Research group, Liverpool School of Tropical Medicine, Liverpool, UK; 2 Health Protection Agency, Porton Down, Salisbury, UK; 3 Department of Clinical Infection, Microbiology and Immunology, University of Liverpool, Liverpool, UK; 4 Gene Technology Unit, Department of Genetic Medicine, Women's and Children's Hospital, Adelaide, Australia; 5 Vaccine Vector Group, Dept of Virology, Imperial College London, London, UK; 6 Institute of Cellular Medicine, Newcastle University, UK; 7 Consultant in Infectious Diseases & Tropical Medicine, Department for Infectious Diseases, North Manchester General Hospital, Delaunay's Road, Manchester M8 5RB, UK

**Keywords:** Prime-boost HIV vaccine, broadly reactive neutralising antibodies, recombinant poxvirus, modified vaccinia virus Ankara, fowlpox virus, cholera toxin B, human complement protein C3d, virus-like particle

## Abstract

**Background:**

There is renewed interest in the development of poxvirus vector-based HIV vaccines due to the protective effect observed with repeated recombinant canarypox priming with gp120 boosting in the recent Thai placebo-controlled trial. This study sought to investigate whether a heterologous prime-boost-boost vaccine regimen in Chinese cynomolgus macaques with a DNA vaccine and recombinant poxviral vectors expressing HIV virus-like particles bearing envelopes derived from the most prevalent clades circulating in sub-Saharan Africa, focused the antibody response to shared neutralising epitopes.

**Methods:**

Three Chinese cynomolgus macaques were immunised via intramuscular injections using a regimen composed of a prime with two DNA vaccines expressing clade A Env/clade B Gag followed by boosting with recombinant fowlpox virus expressing HIV-1 clade D Gag, Env and cholera toxin B subunit followed by the final boost with recombinant modified vaccinia virus Ankara expressing HIV-1 clade C Env, Gag and human complement protein C3d. We measured the macaque serum antibody responses by ELISA, enumerated T cell responses by IFN-γ ELISpot and assessed seroneutralisation of HIV-1 using the TZM-bl β-galactosidase assay with primary isolates of HIV-1.

**Results:**

This study shows that large and complex synthetic DNA sequences can be successfully cloned in a single step into two poxvirus vectors: MVA and FPV and the recombinant poxviruses could be grown to high titres. The vaccine candidates showed appropriate expression of recombinant proteins with the formation of authentic HIV virus-like particles seen on transmission electron microscopy. In addition the b12 epitope was shown to be held in common by the vaccine candidates using confocal immunofluorescent microscopy. The vaccine candidates were safely administered to Chinese cynomolgus macaques which elicited modest T cell responses at the end of the study but only one out of the three macaques elicited an HIV-specific antibody response. However, the antibodies did not neutralise primary isolates of HIV-1 or the V3-sensitive isolate SF162 using the TZM-bl β-galactosidase assay.

**Conclusions:**

MVA and FP9 are ideal replication-deficient viral vectors for HIV-1 vaccines due to their excellent safety profile for use in humans. This study shows this novel prime-boost-boost regimen was poorly immunogenic in Chinese cynomolgus macaques.

## Background

The development of a safe, affordable and effective HIV-1 vaccine remains a priority especially in sub-Saharan Africa where the hypervariability of the virus poses the greatest challenge. While numerous HIV-1 vaccine candidates have been developed, only three HIV-1 vaccine regimens have been tested in Phase III clinical trials for efficacy: VaxGen's AIDSVAX gp120 vaccine induced non-neutralising antibodies which failed to provide protection to immunised individuals [1]; the STEP vaccine regimen comprised 3 recombinant adenovirus serotype 5 viruses expressing HIV-1 Gag, Pol and Nef, that induced CD8^+ ^T cell responses to viral antigens but afforded no protection to vaccinees [2,3]; and the recent Thai placebo-controlled trial of repeated recombinant canarypox virus priming with recombinant gp120 boosts was designed to give antibody rather than T cell responses. A post-hoc modified analysis showed modest efficacy in preventing HIV-1 infections [4], but the placebo arm did not incorporate a poxvirus control to allow for the effects of repetitive stimulation on innate immunity, and no antibody responses capable of neutralising primary isolates of HIV-1 were demonstrated.

Modified vaccinia virus Ankara (MVA) and attenuated fowlpox virus (FPV, particularly strain FP9) are poxviruses that have been safely administered to humans [5-7] as they are replication-defective in human cells [8,9]. In addition, the vectors have no apparent restriction in the quantity of additional recombinant DNA they can accommodate and can be grown to high titres in chick embryo fibroblasts (CEFs). Moreover, the recombinant poxvirus vaccine stocks are stable at room temperatures for extended periods of time without significant losses in titre and indefinitely if the poxvirus is immobilised onto carbohydrate glass [10]. Recombinant MVA (rMVA) and recombinant FPV (rFPV) have been developed as HIV-1 vaccine candidates and tested in heterologous prime-boost combinations with DNA vaccines in mice [11-13], macaques [14-18] and humans [19-24]. These vaccine approaches principally elicit cytotoxic T lymphocyte (CTL) responses which are thought to be an important component of protective immunity to HIV-1 (reviewed in [25]). In the original prime-boost CTL work it was found that T cell responses were selectively boosted to epitopes held in common by the priming and boosting agents [26,27]. The mechanism of the boosting is thought to be due to type-1 interferon production stimulated by MVA (MVA lacks the soluble interferon receptors that might block this): i.e. the adaptive immune response is being boosted by the innate immune response [8]. An important limitation of T cell-based vaccines is that they may not achieve sterilising immunity to HIV-1, but instead will hopefully control virus replication [28], so there has been a considerable focus on the development of immunogens that can elicit both T cell immunity and a broadly-reactive neutralising antibody (NAbs) response to HIV-1 [29-31]. However, the design immunogens that elicit NAbs that neutralise a broad range of primary isolates is proving to be particularly challenging [32]. Nevertheless, NAbs have been identified in chronically infected individuals that have potent neutralising activity and monoclonal antibodies (MAbs) have been generated from these donors (reviewed in [32,33]. The epitopes targeted by these antibodies have been characterised and serve as templates for candidate HIV-1 vaccines [34,35]. The data from the VAXGEN and STEP studies using soluble gp120 and vectored Env immunogens showed the antibodies elicited were reactive with only a small subset of V3-sensitive isolates of HIV-1 [1-3]. Thus, the design of improved Env immunogens remains a major goal to HIV-1 vaccinologists.

The best protection in non-human primates (NHPs) is obtained by prior exposure to attenuated lentiviruses where the immune correlates of protection remain ill-defined [36] reviewed in [37]. Currently, a live attenuated HIV-1 vaccine for humans is beset by safety concerns due to the risks associated with mutation and reversion to a wild-type virulent form [38]. A safe and promising approach is to generate HIV virus-like particles (VLPs) bearing authentic Env trimers, which are a highly effective form of subunit vaccine that mimic the antigenic structure and size of a virus particle but lack genetic material so are non-infectious (reviewed in [39]. VLPs are often highly immunogenic and thus there has been considerable interest in using this approach so a number of VLP forming HIV vaccine candidates have been described [14,40-48] with some of the vaccines progressing to clinical trials [20]. Some of these studies employed a heterologous DNA prime, recombinant rMVA boost [14,20,40,43,44,47]. However, the antibodies elicited are often focused on non-functional forms of Env, possibly gp120/41 monomers and not on functional Env trimers [41] so there is a need to ensure that the B cells are recognising functional forms of Env. In addition, vaccine candidates will need to provide potent immunostimulatory signals to the relevant B cells so that high titres of NAbs are obtained. Another advantage of recombinant VLP forming vaccine candidates is that they can express additional proteins such as immunostimulatory proteins to potentiate NAbs.

Adjuvants are increasingly incorporated into the design of vaccines to further improve their immunogenicity (reviewed in [49,50]). The co-administration of adjuvants necessitates continuous refrigeration, so coencoding adjuvants bypasses this need and simplifies vaccine administration in resource-limited settings. In this study we sought to overcome the poor immunogenicity of cross-clade neutralising epitopes and the generally poor antibody responses of DNA prime, poxvirus boost regimens by the incorporation of the B cell adjuvant human complement protein C3d (hC3d) [51,52] in the rMVA vaccine candidate and the mucosal adjuvant non-toxic cholera toxin B (CTB) in the rFPV vaccine candidate. The adjuvants effects of murine C3d have been shown to boost antibody responses in mice when coexpressed with DNA vaccines expressing Env [53-55]. Cholera toxin has been widely used an adjuvant (reviewed in [56], and has been shown to enhance antibody responses when co-administered with recombinant vaccinia [57] and MVA [58]. In this investigation, only the B subunit was used because of the toxicity associated with the use of the cholera holotoxin, and because the related E.coli holotoxin has been linked with facial palsy when co-administered with influenza VLPs [59].

In this study, a novel prime-boost-boost vaccination regimen was assessed for immunogenicity following vaccination of Chinese cynomolgus macaques. Each vaccine vector expressed a different clade of Env (clades A, D and C). HIV VLPs were used to deliver the Env to the immune system as authentic Env trimers. To further enhance the immunogenicity of Env we have coencoded B cell adjuvants into the recombinant poxviral vectors. We proceeded directly to a NHP model of immunogenicity because this model most closely resembles the likely immune response in humans and given the fact that certain neutralising monoclonal antibodies are known to be polyreactive to self antigens [60,61] this avoids false positive results from murine studies. Furthermore, the effectiveness of hC3d is most likely to be demonstrated in a NHP model.

## Results

### DNA vaccine

**Figure 1:**
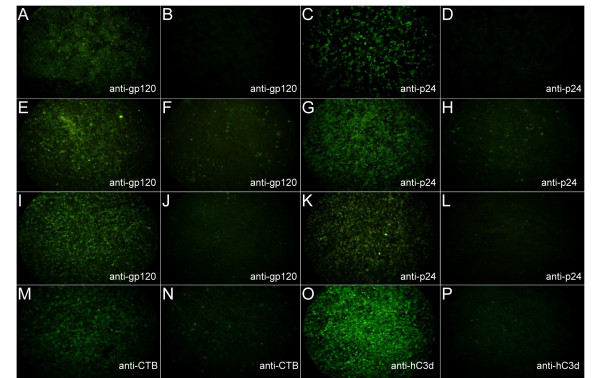
**Detection of recombinant HIV-1 proteins and adjuvants using immunofluorescent microscopy**. HEK293 cells stained with: (A) anti-gp120 post-tranfection with DNA vaccine; (B) anti-gp120 with lipofectin control; (C) anti-Gag post-transfection with DNA vaccine; (D) anti-Gag with lipofectin control. Chick embryo fibroblasts stained with: (E) anti-gp120 post-infection with rFPV; (F) anti-gp120 post-infection with wild-type FPV; (G) anti-Gag post-infection with rFPV; (H) anti-Gag post-infection with FPV; (I) anti-gp120 post-infection with rMVA; (J) anti-gp120 post-infection with wild-type MVA; (K) anti-Gag post-infection with rMVA; (L) anti-Gag post-infection with wild-type MVA; (M) anti-CTB post-infection with rFPV; (N) anti-CTB post-infection with wild-type FPV; (O) anti-hC3d post-infection with rMVA and (P) anti-hC3d post-infection with wild-type MVA. Magnification ×50.

DNA plasmid encoding consensus HIV clade A *env *was shown to express gp120 by immunofluorescence studies on transfected HEK293 cells (Figure 1A). DNA plasmid encoding HIV clade B *gag *was shown to express Gag protein by immunofluorescence studies on transfected HEK293 cells (Figure 1C), as previously reported [62]. In all cases specific MAbs were used with appropriate lipofectin only controls (Figure 1B and 1D).

### Recombinant poxvirus HIV vaccines

The rFPV infected CEFs were shown to express HIV Env (Figure 1E), HIV Gag (Figure 1G) and CTB (Figure 1M) by immunofluorescence. In addition, CEFs infected with rMVA were shown to express gp120 (Figure 1I), Gag (Figure 1K) and hC3d (Figure 1O) using immunofluorescence. In all cases specific MAbs were used with appropriate non-recombinant controls (Figure 1).

### HIV-1 neutralising epitopes

**Figure 2:**
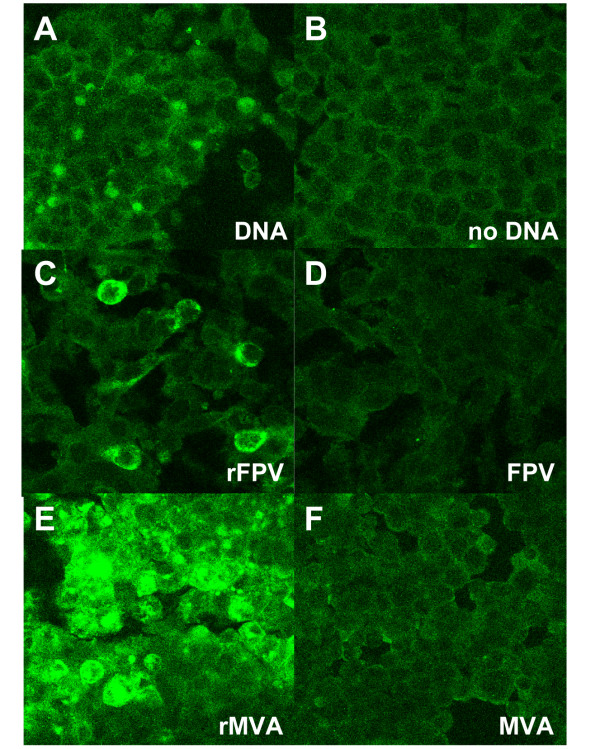
**Detection of a b12 epitope expressed by HIV-1 vaccine candidates**. Staining of HEK293 cells with IgG1b12 post-transfection/infection with HIV vaccine candidates: (A) post-transfection with clade A Env expressing DNA vaccine; (B) lipofectin control; (C) post-infection with rFPV; (D) post-infection with wild-type FPV; (E) post-infection with rMVA; (F) post-infection with wild-type MVA. Magnification ×200.

The b12 neutralising epitope was demonstrated to be present on the surface of transfected/infected HEK293 cells for all 3 vaccine candidates using confocal immunofluorescent microscopy (Figure 2), with strongest staining for b12 seen for rMVA infected cells (Figure 2E), with less so for rFPV infected (Figure 2C) and DNA transfected cells (Figure 2A). In all cases MAb b12 was used with appropriate non-recombinant/lipofectin only controls (Figure 2B, D and 2F). Anti-gp120 MAb 2G12 and anti-gp41 MAb 2F5 were shown to not bind to all recombinant infected/transfected cells under the assay conditions employed (data not shown).

### VLP formation

**Figure 3:**
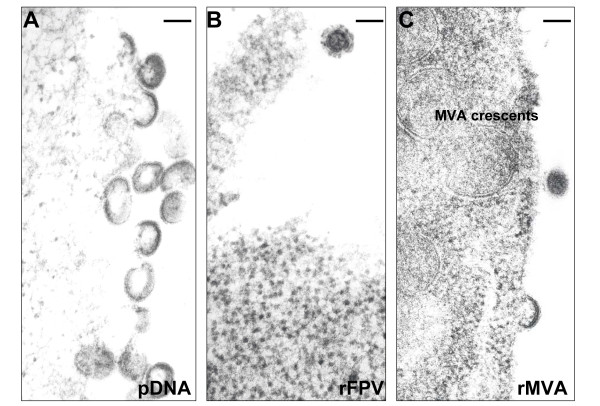
**HIV VLP secretion by HEK293 cells infected/transfected with vaccine candidates as revealed by TEM: (A) post-transfection with dual plasmid DNA vaccine candidate**. Magnification × 100,000; (B) post-infection with rFPV. Magnification × 100,000; (C) post-infection with rMVA. Magnification × 100,000. Uninfected HEK293 cells were screened by TEM for virus particles but no viruses were observed in any grids (data not shown). Bar = 120 nm.

All three vaccine candidates were shown to produce HIV virus-like particles on TEM of transfected/infected human-derived HEK293 cells (Figure 3 and Additional File 1, Figure S1). HIV VLP production was prolific in the case of rMVA (Figure 3C and Additional File 1, Figure S1C), but much less for rFPV (Figure 3B and Additional File 1, Figure S1B). The dual DNA vaccine produced large numbers of VLPs (Figure 3A and Additional File 1, Figure S1A) from transfected cells but the efficiency of transfection limited the number of VLP producing HEK293 cells. No VLPs were seen on inspection of non-transfected or uninfected HEK293 cells (data not shown), indicating that VLP production seen with vaccine candidates was specific.

### Immunisation studies

All three animals were vaccinated simultaneously following an identical schedule using the same batches of vaccine candidates. No adverse events were reported on vaccination of macaques.

### Immunogenicity studies

**Figure 4:**
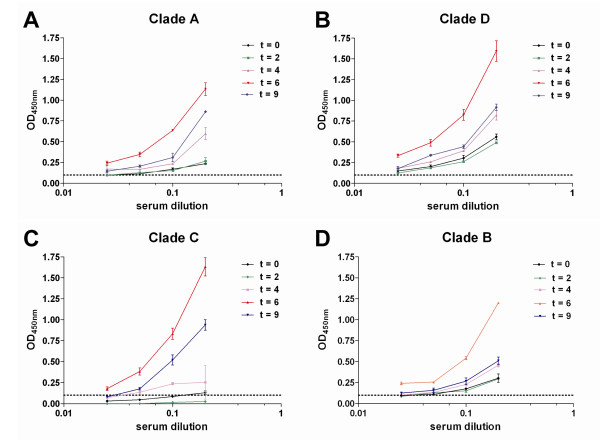
**Antibody responses to HIV-1**. Antibody responses from vaccinated macaque 1057 in serum samples collected at: week 0 (black line); week 2 (green line); week 4 (mauve line); week 6 (red line) and week 9 (blue line) to primary isolates of HIV-1/SF162 as measured by ELISA. Serum antibody responses to: (A) clade A 92/UG/037; (B) clade D 94/UG/114; (C) clade C 97/ZA/003; (D) SF162. Determinations were duplicated and represented as the mean of 2 tests ± SEM.

We first assessed HIV-specific antibody responses elicited by the cynomolgus macaques following the prime-boost-boost vaccinations by ELISA using inactivated HIV-1 virions (clades A, D, C and B) as the antigen. Serum antibodies were measured over the complete time course of the study (t = 0, 2, 4, 6 and 9 weeks). The immunisation regimen elicited HIV-specific antibodies in macaque 1057 (Figure 4). The antibody response peaked at week 6 which was 2 weeks after the macaques had been vaccinated with the rMVA vaccine candidate but the antibody responses were short lived as it was much lower by week 9. The highest antibody responses were generated to primary isolates of HIV clades D and C (Figure 4B and 4C). No anti-HIV antibodies were detected in macaques 9035 and 2027.

**Figure 5:**
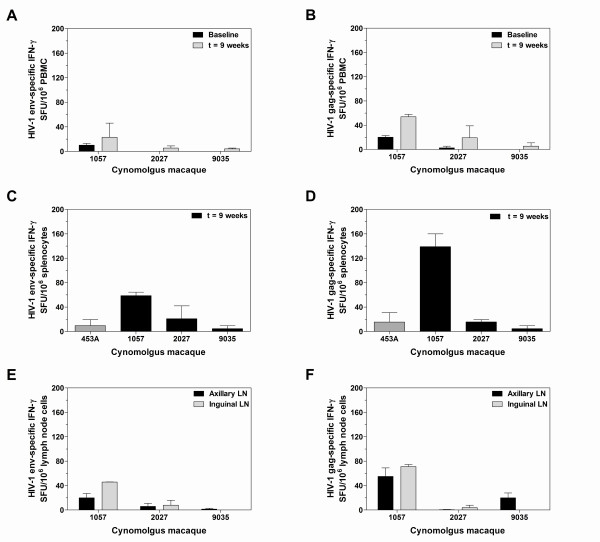
**HIV-specific T cell responses by IFN-γ ELISpots at week 9**. (A) Pre-immunised and post-immunised Env-specific peripheral blood mononuclear cell (PBMC) responses; (B) pre-immunised and post-immunised Gag-specific PBMC responses; (C) Env-specific splenocyte responses; (D) Gag-specific splenocyte responses; (E) Env-specific axillary and inguinal lymph node responses and (F) Gag-specific axillary and inguinal lymph node responses. Data is duplicated and presented as the mean number of spot-forming units/10^6 ^cells ± SEM.

We next assessed the magnitude of T cell responses elicited in the immunised macaques against overlapping peptide pools from either HIV-1 Gag (p24/p17) or Env gp120 in the *ex vivo *IFN-γ ELISpot assay (for amino acid sequences see Additional File 2, Table S1). Macaque 1057 showed a moderate naive PBMC response to Env peptides. All macaques elicited a positive PBMC response to Env peptides at the end of the time course (Figure 5A). Small preimmunised PBMC responses to Gag peptides were detectable in macaques 1057 and 9035. All macaques elicited a positive PBMC response to Gag peptides at week 9 (Figure 5B). Splenocyte responses were clearly seen in response to peptides from both Env and Gag in macaque 1057 (Figure 5C and 5D). Macaques 2027 and 9035 elicited a similar splenocyte response to Gag and Env peptides to the naïve macaque 453A (Figure 5C and 5D). Positive T cells responses from both axillary and inguinal lymph nodes were observed in all macaques but the strongest T cell responses were found in macaque 1057 (Figure 5E and 5F).

**Figure 6:**
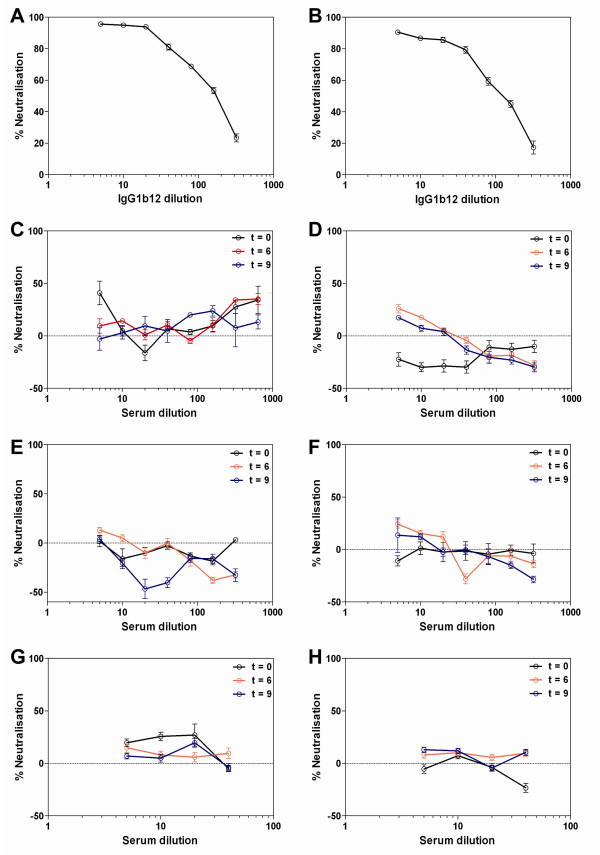
**HIV-1 neutralisation assays**. (A) Potency of IgG1b12 at neutralising lab-adapted isolate SF162, 1:5 IgG1b12 = 20 μg/mL; (B) potency of IgG1b12 at neutralising primary clade B isolate 91/US/005, 1:5 b12 = 20 μg/mL; Macaque serum collected at 3 time points: Solid black line = week 0; solid blue line = week 6; solid red line = week 9. Macaque 1057 seroneutralisation of primary isolates: (C) clade A isolate 92/UG/037; (D) clade D isolate 94/UG/114; (E) clade C isolate 97/ZA/003; (F) lab-adapted clade B isolate SF162; (G) human complement/macaque serum and clade C isolate 97/ZA/003; (H) heat inactivated human complement/macaque serum and clade C isolate 97/ZA/003. Data are presented as the mean of 3 tests ± SEM.

We next assessed whether the HIV-specific antibody response detected in macaque 1057 would neutralise primary isolates of HIV-1 using the TZM-bl cell neutralisation assay. The assay was validated by the detection of potent neutralisation of SF162 by IgG1b12 (Figure 6A), yielding similar concentrations to those previously reported to achieve 90% and 50% neutralisation of SF162 [63]. Furthermore, there was neutralisation of a clade B primary isolate of HIV-1 (91/US/005) by IgG1b12 (Figure 6B) and a clade C primary isolate of HIV-1 (97/ZA/003) using the gp41 MAb 4E10 (see Additional File 3, Figure S2). The neutralising activity of serum from macaque 1057 was tested at baseline, week 6 and week 9. We report here that no neutralising antibodies were detectable in the serum of macaque 1057 at any of the time points through the time course of the study. Representative HIV neutralisation assays obtained from macaque 1057 are shown (Figure 6). There was no HIV neutralisation when serum from macaque 1057 was cultured in the presence of primary HIV clade A isolate 92/UG/037 (Figure 6C), clade D isolate 94/UG/114 (Figure 6D), clade C isolate 97/ZA/003 (Figure 6E) and the b12 sensitive strain SF162 (Figure 6F). Furthermore, there was no detectable neutralisation of 97/ZA/003 when the macaque serum was mixed with human complement (Figure 6G and 6H). We also looked for NAbs in the sera of macaques with no apparent humoral immune response, but as expected these were negative.

## Discussion

This study shows that large and complex synthetic DNA sequences can be successfully cloned in a single step into two poxvirus vectors: MVA and FPV and recombinant poxviruses could be grown to high titres without the recombinants reverting to their wild-type form. The vaccine candidates showed appropriate expression of recombinant proteins in infected/transfected cells and the b12 epitope (coincident with the CD4 binding site (CD4bs) of gp120) was shown to be held in common by the vaccine candidates. The CD4bs is an important target for NAb responses identified in HIV-1 infected individuals [64]. In addition human cells infected/transfected with the vectors showed expression of authentic HIV-like VLPs. The HIV vaccine candidates were delivered by intramuscular injection of Chinese cynomolgus macaques in a prime-boost-boost vaccination protocol. The vaccines were tolerated without any adverse reactions. The vaccines elicited modest T cell responses in the immunised macaques but only macaque 1057 produced an HIV-specific antibody response which was highest after the third heterologous immunisation. However, the antibodies did not neutralise the panel of primary HIV isolates or the laboratory adapted, b12-sensitive isolate SF162 using the TZM-bl β-galactosidase assay. The TZM-bl neutralising antibody readout has been validated against protection from SHIV infection in passive transfer experiments [65]. Our immunisation protocol was shorter than those generally used for subunit vaccines aimed at eliciting antibody responses but in keeping with those used for heterologous prime-boost aimed at eliciting T cell responses. Antibody responses capable of neutralising SHIV are generally apparent after the second subunit boost [66], but in natural HIV infection it can take some time to emerge [67-69]. We detected no evidence of NAb responses 5 weeks after the third heterologous immunisation.

The vaccine candidates directed VLP secretion from infected/transfected cells *in vitro*, however, we have not demonstrated VLP production following vaccination *in vivo*: a difficult subject to study without biopsying vaccination sites. The rMVA produced a prolific number of VLPs from infected HEK293 cells compared to the DNA and rFPV vaccines. Recombinant proteins in MVA were expressed from combination early/late promoters (both being effective in mammalian cells) whereas the recombinant proteins in FPV were expressed from early promoters alone (only early promoters being effective in FPV-infected mammalian cells). We have not proved that Env is incorporated in the membranes of the VLPs, although the appearance of Env spikes on TEM is highly suggestive. Others have also reported expression of the b12 epitope on Gag-Env pseudovirions [70] but not in the context of carriage by poxviruses. Expression and VLP formation from the plasmid constructs used in the DNA vaccine would probably have been enhanced if a single plasmid expressing both Env and Gag were used, but we were unable to obtain such materials. The Env expression plasmid employed is *rev*-independent. We used codon-optimised *env *consensus sequences for clades A and C which are known to be functional and CCR5-using. No consensus sequence for clade D *env *was available at the time, so we derived a codon-optimised version from the CCR5-using infectious molecular clone U88824. Functional consensus sequences were used where possible because these are believed to enhance NAb responses [71].

The reason for the failure to generate NAbs is not clear. It may be that the vectors employed simply do not generate good antibody responses despite the attempts to improve this with hC3d and CTB. The hC3d was incorporated towards the N-terminus of Env (essential if it is to be displayed on the external surface of a VLP) whereas the original work in rodents with hen egg lysozyme emphasised the importance of incorporation at the C terminus [51]. Furthermore, most reports describe the use of murine C3d as molecular adjuvants [51,72] but here we used hC3d because we reasoned it was more relevant for human vaccine development and our NHP model. In addition, we have not used triplet sequences of hC3d because highly repetitive sequences are rapidly deleted by poxviruses, and we predicted the trimeric structure of HIV Env would perform this function naturally anyway. The approach of using C3d as a molecular adjuvant in recombinant viral vectors has recently been shown to hamper antibody responses to certain antigens [73] and this study suggests that encoding C3d was counterproductive to the vector design. CTB was preferentially expressed in FPV not MVA, because MVA is known to block the effect of interleukin-1β [8] by production of a soluble receptor, and this would likely interfere with the adjuvant effect of CTB [74]. Furthermore, the CTB was designed to be secreted from poxvirus-infected cells with no fusion with candidate HIV antigens. We have not proved that the CTB and hC3d expressed by the poxviruses are functional.

Since these experiments were conceived it has also become apparent that the native b12 epitope is a poor immunogen: it is located deep in the CD4bs, so the b12 MAb has an unusually extended variable loop in order to bind the epitope. Studies suggest that steric hindrance, e.g. by beta20-beta21 loop [75], would prevent good immune responses to this epitope in a similar fashion to steric hindrance of any coreceptor binding site epitopes [76]. Furthermore, naturally glycosylated HIV-1 Env trimers are poor immunogens, so it is possible that further modifications to the Env amino acid sequence in order to better expose neutralising epitopes might be beneficial in addition to the cross-clade immunisation employed here.

Although we have focused on the b12 epitope it is quite possible that there were other cross-clade neutralising epitopes present in the vaccine candidates, whether on gp120 or gp41. For example, the highly conserved caveolin-binding motif (^623^WNNMTWMEW^631^) of gp41 is represented in the amino acid sequence of all the constructs [77,78], although this does not appear to be immunogenic except when expressed in isolation. The TZM-bl β-galactosidase assay we employed would be expected to detect the effect of any antibody such as the gp120 MAb IgG1b12 that interfered with HIV-CD4 binding, HIV-coreceptor binding or fusion of HIV Env and target cell membrane. It is known that certain antibody subpopulations such as 2G12-like antibodies, may not be detected through the use of the TZM-bl assay [79] and that high levels of CCR5 expression can reduce sensitivity for antibodies such as 4E10 [80]. Even so, this assay is the most standardised and widely applied assay for the measurement of neutralising antibodies [65,80,81] and alternative formats such as PBMC-based assays show great variability in sensitivity in inter-laboratory comparisons [80].

T cell responses were clearly seen on ELISpots to conserved Gag and Env peptides in the macaques at the end of the study (five weeks after the final rMVA immunisation). This finding is consistent with previous studies in cynomolgus macaques using DNA prime, MVA boost regimens [82,83]. T cell responses in DNA prime, poxvirus boost regimens generally peak earlier than this at around 1 week post-immunisation [27], so it is possible that more vigorous T cell responses have been missed. It may also be the case that cross-clade T cell responses in macaques may not translate to humans, because the T cell epitopes are different and many are clade-specific.

Of note both antibody and T cell responses were best in the heaviest macaque 1057, the other two macaques were significantly smaller (see Additional File 4, Table S2). There was no obvious pathology at post mortem in any of the macaques. HIV and SIV vaccine candidates have not been extensively studied in Chinese cynomolgus macaques, and there is no data on MHC types, so future investigations may be better performed in the rhesus macaque model.

In conclusion, FPV and MVA are ideal replication-deficient viral vectors for HIV-1 vaccines due to their excellent safety profile for use in humans. This study shows that the DNA and poxvirus vectors used according to the immunisation protocol were poorly immunogenic in Chinese cynomolgus macaques. Furthermore, the antibodies elicited in the macaque did not neutralise primary or lab adapted isolates of HIV-1. Clearly it is very difficult to prove a negative result, and we cannot exclude the possibility that the viral vectors may elicit NAbs in combination with other vaccine candidates or in different model systems (e.g. Rhesus macaques), or with modifications to the vaccine vectors or adjuvants. The level of VLP production by the MVA recombinant was prolific, and this rMVA vaccine candidate may be worth revisiting with DNA and FPV vaccine candidates that are equally prolific producers of VLPs. We draw attention to the fact that we have published comprehensive sequence data and we will make our reagents available to bona fide vaccine researchers who wish to explore these issues.

## Methods

### Cell lines

The HEK293 kidney cell line was obtained from the European Collection of Cell Cultures (ECACC, Salisbury, UK). CEFs were obtained from 9-10 day old embryonated eggs from specific pathogen free Rhode Island Red chickens (Institute of Animal Health, Newbury, UK). Human peripheral blood mononuclear cells (PBMCs) were obtained as leucopaks (Merseyside Blood Transfusion Service, Liverpool, UK) and from healthy donors. TZM-bl cells [84,85] were obtained from the NIH AIDS Reference and Reagent Program (NIH ARRRP; Germantown, US).

### DNA vaccine

Two DNA expression vectors used for immunisation were codon-optimised for human expression. A plasmid (pcDNA3.1-A consensus gp160-opt) encoding HIV clade A consensus gp160 (GenBank: HM070998) under a CMV immediate early promoter was obtained from Beatrice Hahn and the other plasmid (pHCMVwhvgagml) encoding HIV clade B *gag *(codon-optimised for human expression) under a CMV early promoter was obtained from Don Anson. The clade B *gag *sequence was derived by Don Anson from the published sequence data for HIV-1 strain YU2 (GenBank: M93258). Plasmid DNA for injections was purified on anion exchange columns (Endotoxin-free Plasmid Maxi kit, QIAGEN, Crawley, UK) and diluted in endotoxin-free saline (0.9% w/v NaCl).

### Recombinant FPV vaccine

FPV strain FP9 was used. Open reading frames for full length codon-optimised HIV-1 clade D *gag*, *env *and CTB (fused to poxvirus leader sequence) were arranged on a single stretch of DNA with synthetic back-to-back early poxviral promoters driving the HIV components (GenBank: HM070999). The HIV-1 clade D *gag *and *env *amino acid sequence was derived directly from the infectious molecular clone U88824. This DNA was synthesised *de novo *(Blue Heron Biotechnology, Bothell, USA); the open reading frames were not entirely codon-optimised because some bases were changed to reduce predicted RNA secondary structure. Certain unique restriction sites were preserved; poxvirus termination sequences and the ribosomal slippage site were mutated. The synthetic sequence was flanked by *NgoM*IV sites, which were used for subcloning into the *Xma*I (*Sma*I) site of the pEFL29 recombination vector. Correct orientation of the insert was necessary so that CTB subunit production would be driven by an existing promoter in pEFL29.

### Recombinant MVA vaccine

MVA from human smallpox vaccine stock (obtained from Anton Mayr) was used. Open reading frames for full length consensus codon-optimised clade C *gag *and *env *were arranged on a single stretch of DNA with synthetic back-to-back early/late poxviral promoters driving the HIV components. The sequence for monomeric hC3d (cynomolgus macaque C3d gene sequence is not available, rhesus macaque C3d shows 95% amino acid sequence homology to hC3d) was inserted just after the *env *leader sequence, with intervening Gly/Ser spacer polypeptide sequence. The active site Cys codon of C3d was mutated to Ser. The *env *sequence was further modified to enhance gp41/gp120 cleavage by incorporation of six Arg residues at the furin cleavage site, and a disulphide bridge was introduced to link gp41 and gp120 by mutating the Ala 480 codon and Thr 584 codon to Cys codons. (GenBank: HM071000). This DNA was synthesised *de novo *(Blue Heron Biotechnology); the open reading frames were not entirely codon-optimised because some bases were changed to reduce predicted RNA secondary structure. Certain unique restriction sites were preserved; poxvirus termination sequences and the ribosomal slippage site were mutated. The synthetic sequence was flanked by *NgoM*IV sites which were used for subcloning into the *Xma*I (*Sma*I) site of the pSC11 recombination vector (obtained from Geoffrey Smith).

### Verification of recombinants

Recombinant virus was isolated using β-galactosidase substrate X-gal (5-bromo-4-chloro-3-indoyl-D-galactopyranoside) soft-agar overlay of infected CEF monolayers (MVA, [86]; FPV, [87]). Plaque-purification was performed six times on CEFs prior to large-scale virus propagation and purification on sucrose cushions. Purity and titre of poxvirus recombinants were checked by plaque assay on primary CEFs under soft agar with an X-gal overlayer.

### Confirmation of protein expression

Expression of recombinant proteins was demonstrated by immunofluorescence of infected CEFs/transfected HEK293 cells using the following MAbs: anti-gp120 (NIH ARRRP) [88]; anti-p24 (NIH ARRRP) [89]; anti-CTB (Abcam, Cambridge, UK); and anti-human C3d (Abcam) followed by detection with appropriate secondary antibodies. Expression of neutralising epitopes was shown by confocal immunofluorescence of infected/transfected HEK293 cells using the anti-gp120 MAb IgG1b12 [90,91], anti-gp120 MAb 2G12 [92] and anti-gp41 MAb 2F5 [93](NIH ARRRP) followed by detection with appropriate secondary antibodies.

### Electron microscopy of HIV VLPs

HEK293 cells were co-transfected with 2.5 μg of each plasmid and incubated for 48 hours at 37°C with 5% CO_2_. HEK293 cells were infected with recombinant poxvirus vaccine candidates at a multiplicity of infection (MOI) of 5 and 50 and incubated for 24-48 hours at 37°C with 5% CO_2_. HEK293 cells were washed and fixed in 2.5% glutaraldehyde in 0.1 M sodium cacodylate buffer (pH 7.3) for 1 hour. Samples were washed twice with phosphate buffered saline (PBS) and resuspended in 2.5 mL of 50% ethanol and pelleted by centrifugation. The cells were dehydrated in a graded ethanol series and embedded in medium grade LR white embedding resin (SPI, Pennsylvania, US). The resin-embedded tissues were sectioned with an ultramicrotome, stained with 2% uranyl acetate (pH 7) and lead citrate, and the sections were examined using the Jeol CX100 transmission electron microscope and documented on photographic film.

### Cynomolgus macaques

Three male 4-5 year old cynomolgus macaques (*Macaca fascicularis*) were obtained from a Home Office approved breeding colony in China and were acclimatised for two weeks prior to the study commencing. All animals were housed according to the Code of Practice of the UK Home Office (1989) and were sedated with ketamine hydrochloride prior to immunisation and/or venepuncture. All procedures involving animals were approved by the Ethical Review Committee of the Health Protection Agency, UK.

### Immunisations

Macaques were immunised by intramuscular injection over a time course of 9 weeks post-acclimitisation. The DNA vaccine (0.5 mg of each plasmid in saline) was injected into the quadriceps muscle of the left leg, followed by boosting 2 weeks later with rFPV vaccine (2 × 10^8 ^plaque forming units [pfu]) by injection into the quadriceps of the right leg, followed by a further boost 2 weeks later with rMVA vaccine (2 × 10^8 ^pfu) by injection into the biceps muscle of the left arm of each macaque. Whilst under sedation clinical parameters were checked such as body weight, temperature and scoring of lymph node swelling (see Additional File 4, Table S2). Blood was collected prior to each immunisation, then at week 6 and week 9. The immunisation sites were checked for assessment of any adverse reactions.

### ELISA for HIV-specific antibodies

Primary and laboratory-adapted isolates of HIV-1 were quantitated using a p24 ELISA (BIORAD, Hemel Hempstead, UK). Immunolon-4 microtitre plates were coated using 500 ng/well of p24 antigen from the HIV-1 isolates in 100 μL RPMI-1640. The virus was inactivated by the addition of 100 μL of β-propiolactone (VWR, Lutterworth, UK; diluted 1:1000 in PBS) and incubated overnight at 4°C. The plates were incubated at 37°C for 3 hours to hydrolyse the β-propiolactone, washed and blocked with 3% goat serum. Macaque serum was diluted (1:5) in blocking buffer followed by serial doubling dilutions in appropriate wells and incubated at 37°C for 1 hour. The negative control was 15% foetal bovine serum in RPMI-1640. Following a wash, 100 μL of goat anti-macaque IgG-HRP conjugated antibody (diluted 1:25000; AbD Serotec, Kidlington, UK) was added to each well and incubated at 37°C for 1 hour. Following a wash, 100 μL of tetramethylbenzidine (Sigma, Poole, UK) was added and incubated at room temperature in darkness for 30 minutes. The reaction was stopped by the addition of 1N H_2_SO_4 _(Sigma). Absorbances were read at 450 nm. Determinations of duplicate or triplicate tests were averaged ± SEM. Positive antibody responses were defined as twice the background control.

### IFN-γ ELISpots

Mononuclear cells were obtained from peripheral blood and tissue by density gradient centrifugation using standard procedures. Sterile 96-well polyvinylidene difluoride multiscreen plates (Millipore, Billerica, USA) were coated with 100 μL/well of 15 μg/mL GZ-4 coating antibody (MabTech, Nacka Strand, Sweden). Mononuclear cells were plated in duplicate at either: 2 × 10^5 ^and 1 × 10^5 ^cells/well. Following a wash, the cells were incubated with medium alone or with peptide pools (Gag and Env) (NIBSC, Potters Bar, UK). Peptides were either 15 mers or 20 mers and of conserved sequences (see Additional file 2, Table S1) known to be present in the vaccines. Plates were incubated at 37°C with 5% CO_2 _for 16 hours. Following a wash, 100 μL/well biotinylated detector 7-B6-1 antibody (MabTech) diluted to 1 μg/mL in PBS containing 0.5% filtered-FCS was added and incubated at 37°C for 2 hours. Following a wash, Streptavidin-alkaline phosphatase (MabTech) diluted 1:1000 with PBS containing 0.5% FCS was added at 100 μL/well and incubated for 2 hours followed by washing. 100 μL/well of 5-Bromo-4-Chloro-3-Indolyl Phosphate/Nitro Blue Tetrazolium (BCIP/NBT) substrate (Sigma) was added and left at room temperature for 30-60 minutes to allow the reaction to take place producing blue spots around sites of IFN-γ producing cells. After washing, the plates were read and enumerated using an AID ELISpot reader system (Autoimmun Diagnostika GmbH, Straßberg, Germany). Data was analysed by subtracting the mean number of spots in the medium and cells-only control wells from the mean counts of spots in wells with antigen. T cell responses were defined as positive if the number of spot-forming cells were at least twice that of either the naïve macaque control or the preimmunised control.

### Viruses

Primary isolates of HIV-1 including 97/ZA/003, 94/UG/114, 92/UG/037, US/91/005 and SF162 were obtained from the NIH ARRRP. HIV was propagated on PBMC isolated from leucopaks (Merseyside Blood Transfusion Service) employing histopaque (Sigma) density separation followed by stimulation with PHA (Sigma) and IL-2 (NIH ARRRP). High titre supernatants were identified by p24 ELISA using a HIV-1 Ag EIA kit (BIORAD).

### TZM-bl β-galactosidase assay

Neutralisation assays were performed in 96 well, flat-bottomed plates and in triplicate. Wells were seeded with 10^4 ^TZM-bl cells and incubated for 24 hours [84,85]. The TZM-bl cells were treated for 30 minutes with medium containing 2 ng/mL of polybrene (Sigma) and washed with fresh growth medium immediately before the addition of the virus/antibody mixes. HIV was diluted to give 100-200 blue foci per well and mixed with various dilutions of heat inactivated macaque sera or IgG1b12. After incubation for 30 minutes in round-bottom 96 well plates the virus/antibody mixes were transferred onto the TZM-bl cells and incubated for 36-48 hours. Monolayers were fixed briefly with a formaldehyde/glutaraldehyde mix, washed and stained with X-gal solution (4 mM of potassium ferrocyanide, 4 mM potassium ferricyanide, 2 mM MgCl_2_, 0.4 mg/mL X-gal) for 50 minutes. Wells were washed with PBS. Individual wells were photographed and blue foci counted. Data are presented as the percentage of neutralisation in the serum samples compared to the virus-only control ± SEM.

### TZM-bl β-galactosidase assay with human complement

Peripheral blood was taken by venepuncture from normal healthy volunteers and incubated at room temperature until blood was fully coagulated. Serum was collected after centrifugation. Half of the serum was heat inactivated by incubating at 56°C for 90 minutes. HIV isolate 97/ZA/003 was diluted to give 100-200 foci per well. Human sera (heat inactivated or normal serum) was mixed 1:1 with macaque serum and incubated with the diluted HIV. The remaining method is described in the section above.

## List of abbreviations

CD4bs: CD4 binding site; CEFs: chick embryo fibroblasts; CT: cholera toxin; CTB: cholera toxin B; CTL: cytotoxic T lymphocyte; ELISA: enzyme-linked immunosorbent assay; Env: envelope; FCS: foetal calf serum; FPV: fowlpox virus; gp41: glycoprotein 41; gp120: glycoprotein 120; gp160: glycoprotein 160; hC3d: human complement protein C3d; HIV: human immunodeficiency virus; IL-2: interleukin-2; MAbs: monoclonal antibodies; MOI: multiplicity of infection; MVA: modified vaccinia virus Ankara; NAbs: broadly neutralising antibodies; NHP: non-human primate; rFPV: recombinant fowlpox virus; rMVA: recombinant modified vaccinia virus Ankara; PBMC: peripheral blood mononuclear cells; PBS: phosphate buffered saline; PFU: plaque forming unit; PHA: phytohaemagglutinin A; rFPV: recombinant fowlpox virus; rMVA: recombinant modified vaccinia virus Ankara; SFU: spot forming unit; SIV: simian immunodeficiency virus; TEM: transmission electron microscopy; VLP: virus-like particle.

## Competing interests

The authors declare that they have no competing interests.

## Authors' contributions

TJB was the principal investigator, initiated and gained funding for the study, participated in the design and supervision of the project, helped to write the manuscript. SHB designed and performed the majority of experiments, constructed and characterised the recombinant poxviruses, analysed the data, wrote and submitted the manuscript. SAS and MJD coordinated and performed the macaque experiments. SDD performed the ELISpot experiments. BG performed the electron microscopy studies. DSA contributed plasmid DNA expressing clade B Gag. MAS participated in the design of the study and contributed important reagents. JPS participated in the design and supervision of the study and provided laboratory space. All authors have read and approved the manuscript.

## Supplementary Material

Additional file 1 **Figure S1**. HIV VLP secretion by HEK293 cells infected/transfected with vaccine candidates as revealed by TEM: post-transfection with dual plasmid DNA vaccine candidate. *Magnification × 75,000 *(A), post-infection with rFPV. *Magnification × 100,000 *(B), post-infection with rMVA. *Magnification × 60,000 *(C). Uninfected HEK293 cells were screened by TEM for virus particles but no viruses were observed in any grids (data not shown). Bar = 100 nm. Click here for file

Additional file 2 **Table S1**. Amino acid sequences of the overlapping peptide pools used in ELISpot studies. Click here for file

Additional file 3 **Figure S2**. Shows the potency of MAb 4E10 (100 μg/mL) at neutralising the primary clade C HIV isolate 97/ZA/003 using the TZM-bl β-galactosidase assay. Error bars represent the mean of triplicate tests. Click here for file

Additional file 4 **Table S2**. Clinical parameters of the Chinese cynomolgus macaques. Click here for file
