# Supplementary figures and images for: Heterologous prime-boost-boost immunisation of Chinese cynomolgus macaques using DNA and recombinant poxvirus vectors expressing HIV-1 virus-like particles
Source: Virol J. 2011 Sep 7;8:429. doi: 10.1186/1743-422X-8-429 (PMC3177910; doi:10.1186/1743-422X-8-429)

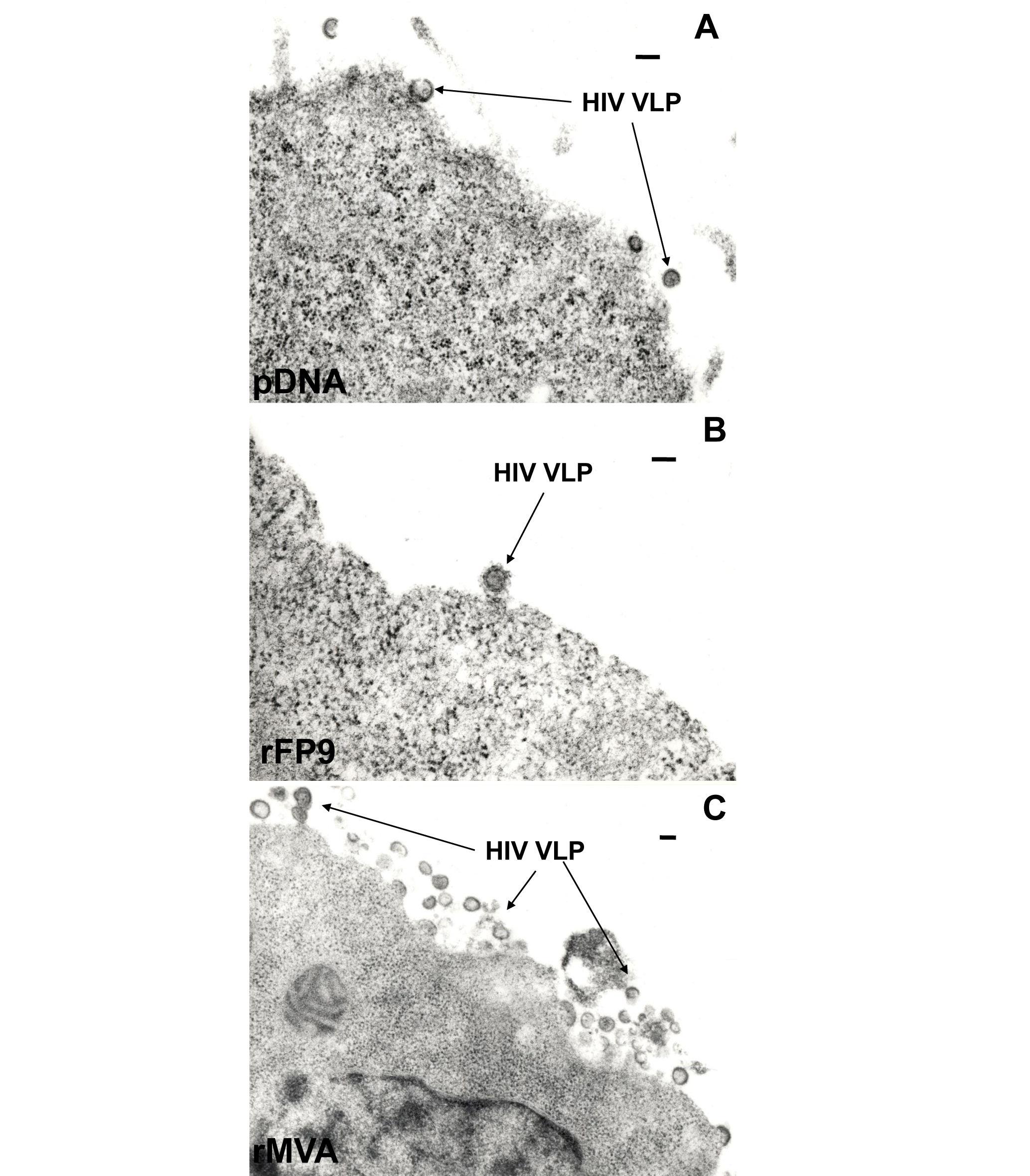

Supplement: Additional file 1 — Figure S1. HIV VLP secretion by HEK293 cells infected/transfected with vaccine candidates as revealed by TEM: post-transfection with dual plasmid DNA vaccine candidate. Magnification × 75,000 (A), post-infection with rFPV. Magnification × 100,000 (B), post-infection with rMVA. Magnification × 60,000 (C). Uninfected HEK293 cells were screened by TEM for virus particles but no viruses were observed in any grids (data not shown). Bar = 100 nm. [file 1743-422X-8-429-S1.TIFF]

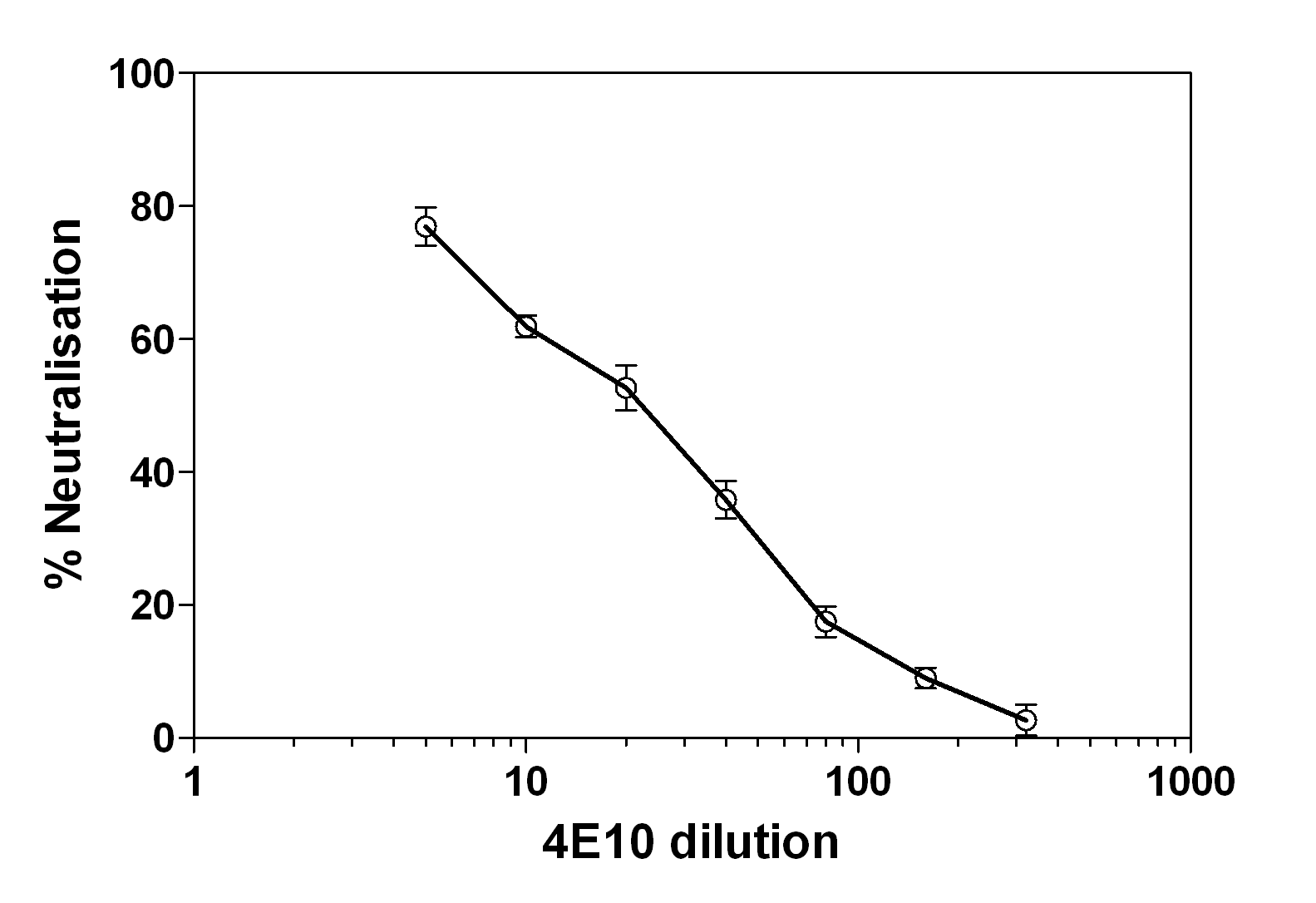

Supplement: Additional file 3 — Figure S2. Shows the potency of MAb 4E10 (100 μg/mL) at neutralising the primary clade C HIV isolate 97/ZA/003 using the TZM-bl β-galactosidase assay. Error bars represent the mean of triplicate tests. [file 1743-422X-8-429-S3.TIFF]
