# Supplementary material for: Heterologous prime-boost-boost immunisation of Chinese cynomolgus macaques using DNA and recombinant poxvirus vectors expressing HIV-1 virus-like particles
Source: Virol J. 2011 Sep 7;8:429. doi: 10.1186/1743-422X-8-429 (PMC3177910; doi:10.1186/1743-422X-8-429)
Supplement: Additional file 2 — Table S1. Amino acid sequences of the overlapping peptide pools used in ELISpot studies. [file 1743-422X-8-429-S2.DOC]

**Additional File 2,** Table S2. A table of amino acid sequences of the overlapping peptides used in the ELISpot studies.
