# Supplementary material for: Heterologous prime-boost-boost immunisation of Chinese cynomolgus macaques using DNA and recombinant poxvirus vectors expressing HIV-1 virus-like particles
Source: Virol J. 2011 Sep 7;8:429. doi: 10.1186/1743-422X-8-429 (PMC3177910; doi:10.1186/1743-422X-8-429)
Supplement: Additional file 4 — Table S2. Clinical parameters of the Chinese cynomolgus macaques. [file 1743-422X-8-429-S4.DOC]

**Additional File 4.**

**Table S2.** A table to show the clinical parameters of the Chinese cynomolgus macaques.

Data are presented as the mean ± SD unless indicated otherwise

α normal range 130-170 g/L

β lymph node score 1 = 1cm
